# Stereoscopic Integrated Imaging Goggles for Multimodal Intraoperative Image Guidance

**DOI:** 10.1371/journal.pone.0141956

**Published:** 2015-11-03

**Authors:** Christopher A. Mela, Carrie Patterson, William K. Thompson, Francis Papay, Yang Liu

**Affiliations:** 1 Department of Biomedical Engineering, The University of Akron, Akron, Ohio, United States of America; 2 John H. Glenn Research Center, Cleveland, Ohio, United States of America; 3 Dermatology and Plastic Surgery Institute, Cleveland Clinic, Cleveland, Ohio, United States of America; University of Pennsylvania, UNITED STATES

## Abstract

We have developed novel stereoscopic wearable multimodal intraoperative imaging and display systems entitled Integrated Imaging Goggles for guiding surgeries. The prototype systems offer real time stereoscopic fluorescence imaging and color reflectance imaging capacity, along with in vivo handheld microscopy and ultrasound imaging. With the Integrated Imaging Goggle, both wide-field fluorescence imaging and in vivo microscopy are provided. The real time ultrasound images can also be presented in the goggle display. Furthermore, real time goggle-to-goggle stereoscopic video sharing is demonstrated, which can greatly facilitate telemedicine. In this paper, the prototype systems are described, characterized and tested in surgeries in biological tissues ex vivo. We have found that the system can detect fluorescent targets with as low as 60 nM indocyanine green and can resolve structures down to 0.25 mm with large FOV stereoscopic imaging. The system has successfully guided simulated cancer surgeries in chicken. The Integrated Imaging Goggle is novel in 4 aspects: it is (a) the first wearable stereoscopic wide-field intraoperative fluorescence imaging and display system, (b) the first wearable system offering both large FOV and microscopic imaging simultaneously, (c) the first wearable system that offers both ultrasound imaging and fluorescence imaging capacities, and (d) the first demonstration of goggle-to-goggle communication to share stereoscopic views for medical guidance.

## Introduction

Surgeons rely on imaging technologies to guide surgical procedures and evaluate the results. Available imaging modalities include Magnetic Resonance Imaging (MRI), X-Ray Computed Tomography (CT) and ultrasound among others [1–7]. Due to their complexity, large size, cost or potential risk associated with long term use these technologies can be difficult to implement in the operating room to guide surgery. Also, it can be difficult to correlate the surgical landscape with the pre-operative images during a surgery [5–7].

When excising a cancerous lesion, the surgeon needs to accurately distinguish between tumor and healthy tissue. Due to the difficulty in applying imaging modalities such as MRI or CT intraoperatively, surgeries are often primarily guided by sight and palpation [8]. Intraoperative ultrasound has been implemented, providing the surgeon with anatomical information and soft tissue contrast. It can, however, still be challenging for the surgeon to correlate the ultrasound imagery with the surgical landscape. When cancerous tissue is not distinguished from healthy tissues, a small fraction of tumors will remain inside the body post operation. This will lead to cancer recurrence and follow-up surgeries. If a positive margin is found, the probability of a local cancer recurrence is high; for example in laryngeal cancer the odds of recurrence go up from 32% to 80% when a positive margin is found [9]. Therefore, accurate margin control is needed.

Traditionally, pathology is the gold standard for margin status determination [8–11]. Pathological analysis requires sectioning of all surgical margins, followed by staining and microscopic investigation, which leads to extensive operating room time. For better surgical margin control, intraoperative optical imaging has emerged as a promising solution [8–12]. Two pertinent approaches have been taken, including large field of view (FOV) imaging and hand-held in vivo microscopy [13–21].

Various large FOV intraoperative fluorescent imaging systems have been developed in the past decade [16–20]. These systems rely upon 2D flat screen displays for relaying fluorescent data to the physician. Fluorescence imaging systems have been developed, offering a quick way to survey the surgical area and guide surgeries [18–20]. However, such systems do not offer the capability of integrated in vivo microscopic imaging. On the other hand, in vivo microscopic imaging systems have been developed [13–15]. These systems hold great potential for in situ pathological analysis. However, the application is limited due to the small FOV and the difficulty involved in surveying all the surgical area in a timely fashion.

More recently, wearable imaging and display systems in a “goggle” form have been developed by Liu et al [22, 23]. These systems have been successfully validated in preclinical and clinical studies [22–24]. Despite encouraging results, these systems only offer 2D imaging and display capabilities, without depth perception. Also, the previous systems are bulky and difficult to wear for longer times. There was also no in vivo microscopy or ultrasound capability offered, limiting the diagnostic power to that of wide-field fluorescence imaging.

To overcome these limitations, herein we report the development of a novel platform technology entitled Integrated Imaging Goggle. The Integrated Imaging Goggle platform is novel in the following aspects:

- It is the first wearable stereoscopic fluorescence imaging system for wide-field intraoperative image guidance. Our system leverages on the principles of stereoscopic vision to present both depth perception and lateral spatial information to the surgeon.
- Our system can image, overlay and present both color reflectance and near infrared florescence information to the user in real time.
- Furthermore, both large FOV fluorescence imaging and handheld microscopic imaging are offered simultaneously. Thus, the surgeon can survey a large area and perform surgeries under the large FOV stereoscopic fluorescence imaging, while investigating suspicious areas in detail with the in vivo microscopic probe.
- The goggle can be integrated with non-optical imaging modalities such as ultrasound, providing multimodal image guidance to the surgeon and mitigating the optical imaging limitation of penetration depth.
- The Integrated Imaging Goggle enables wireless goggle-to-goggle stereoscopic view sharing, where the remote collaborator can visualize the same data that the local goggle wearer sees, with stereovision and depth perception. This is important for remote guidance and telemedicine.

## Materials and Methods

### Imaging System Instrumentation

**Fig 1:**
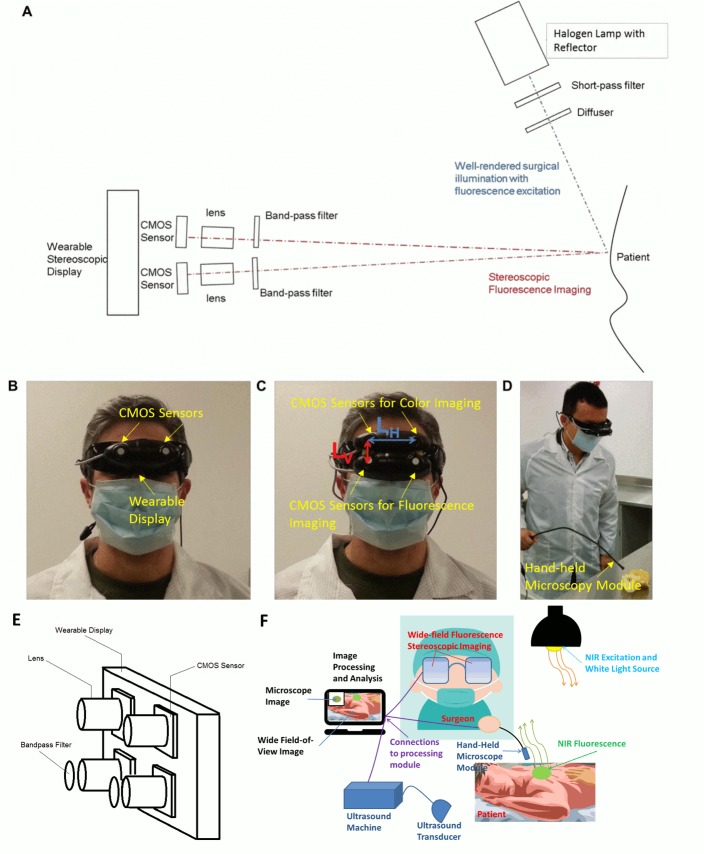
Prototype Integrated Imaging Goggles. (A) Schematic of 2-sensor setup. (B) Photo of 2-sensor setup for stereoscopic fluorescence imaging. (C) Photo of 4-sensor setup for simultaneous stereoscopic color reflectance imaging and fluorescence imaging. Top 2 sensors are for reflectance imaging and bottom 2 sensors are for fluorescence imaging. The horizontal and vertical inter-sensor distances are labeled in blue and red, respectively. (D) Integrated Imaging Goggle with its handheld in vivo microscopy probe. (E) Schematic of 4-sensor setup. (F) Overall system diagram depicting all components and connections during a typical operation with the hand-held microscopy module connected. Alternatively, a portable ultrasound scanner with transducer could be connected to the computational unit.

We have developed two prototype Integrated Imaging Goggle platforms, including a 2-sensor setup (**Fig 1A and 1B**) and a 4-sensor setup (**Fig 1C and 1E**). Both systems are compact, light-weight, and easy to wear. The 2-sensor setup is similar to a previously reported system [25, 26], consisting of 2 complementary metal–oxide–semiconductor (CMOS) imaging sensors housed on a printed circuit board (PCB). Near infrared bandpass filters at 832 nm ±37 nm (OD6) (Edmunds Optics, NJ) were used as emission filters. The second system was a 4-sensor setup for combining color reflectance with fluorescence imaging. It is similar to the 2-sensor setup except 4 imaging sensors are used. The 832 nm bandpass filters for fluorescence imaging were used on 2 of the sensors, while the other 2 unfiltered sensors were used for color reflectance imaging. The prototype systems communicate with laptop computers via USB connections. The light source utilized a 785 nm low-pass filtered (Edmund Optics, NJ) halogen lamp with a glass diffuser which allowed for both white light imaging and infrared excitation of the target, while removing any components near the fluorescent emission bandwidth centered at 830 nm. This way, both well-rendered white light surgical illumination (without NIR component beyond 785 nm) and fluorescence excitation (centered at 780 nm) are achieved concurrently. We further designed and implemented a feature for the light source that enables adjustment of background NIR components (> 830 nm). The background components, in some cases, are helpful in providing references for anatomical structures of the surgical target for the 2-sensor setup. In brief, a small adjustable amount of unfiltered diffuse light from the halogen lamp was allowed to pass from the light box and illuminate the target. This was accomplished by placing a custom lid of the light source on a sliding mechanical stage that could be opened to various levels for allowing in varying amounts of unfiltered light. In addition, the stage could be completely locked shut to eliminate the unfiltered optical component. Furthermore, the Integrated Imaging Goggle has an integrated hand-held microscopic imaging module (Supereyes, Shenzhen, China) with an 832 nm ±37 nm (OD6) bandpass filter (Edmunds Optics, NJ) for in vivo microscopic imaging (**Fig 1D)**. The microscopic module has a resolution of 25 micron. The Integrated Imaging Goggle also has a portable ultrasound scanner (Ultrasonix, China) with B-mode transducer to offer the ultrasound imaging capacity. An overall system diagram is given **(Fig 1F)**.

### Image Acquisition, Processing, Registration and Display

We have developed custom algorithms for real time image acquisition, processing, registration and display. The frames from individual sensors are imported through USB to the computer and synchronized. For the 4-sensor setup, the color reflectance and NIR fluorescence images were merged to create composite images and sent to the wearable stereoscopic display for visualization. In this way, the functional information (fluorescence) was overlaid onto the anatomical information (reflectance). Due to the inter-sensor height disparity between the filtered and unfiltered image sensors (**Fig 1C**), the NIR fluorescence frames had to be aligned to the color reflectance frames. This was accomplished by measuring the vertical inter-sensor distance *L*_*v*_ (25 mm) from the center of sensors as well as the horizontal inter-sensor distance *L*_*h*_ (60 mm) (**Fig 1C**). From this information, a transformation metric, *D*_*V*_, was determined from the equation:

$$\frac{\left(D_{v}+C_{v}\right)}{\left(D_{h}+C_{h}\right)}=\frac{L_{h}}{L_{v}}$$ (1)

where *L* is the measured inter-sensor disparity between sensors in either the horizontal (*H*) or vertical (*V*) direction, *D*_*H*_ is the pixel disparity between common points in the left and right fluorescent images, C_V_ and C_H_ are the calibration correction metrics in the vertical and horizontal directions, respectively. The calibration correction metrics, corresponding to measurable pixel disparities, were manually determined to form a lookup table using fluorescent calibration targets serving as fiducial markers for a given range of working distances (20–40 cm). The points used to calculate *D*_*H*_ were the peak fluorescence points from the calibration targets. A set of calibration metrics was implemented as a lookup table, with the measured pixel disparity as the input, to provide an accurate registration compensating for lateral head movement. So long as the distance to the fluorescent target remains within the range of the calibrated working distance, the registration error was expected to be less than 1 mm. The fluorescence frames were then transformed by the calculated transformation metric *D*_*V*_ so that, after calibration, the fluorescence image representing functional information was aligned to the corresponding color reflectance image representing anatomical information.

Our Integrated Imaging Goggle offers a picture-in-picture display mode when utilizing the hand-held microscope probe. In brief, an additional image frame was added to the top left corner of each goggle’s image frames. This frame displayed the images from the handheld microscope, for close up inspection of the surgical site. When the full frames are viewed stereoscopically through the goggle display screens, the microscope images align over each other and appear as a single 2D image, although the remainder of the frame still appears 3D due to the binocular disparity. A similar process was also implemented for displaying the portable ultrasound images in real time. When operating in ultrasound mode the picture-in-picture display provides the surgeon with subdermal structural information in real time. The ultrasound images are displayed in 2D, similar to the microscope images, while the remainder of the frame still appears in 3D due to the stereoscopic nature of the goggle imaging system. In this way we address part of the limitation of optical imaging for detecting deep tissue structures.

### System Characterization

The Modulation Transfer Function (MTF) for our systems was determined using the slanted edge technique for working distances of 20, 30 and 40 cm from lens to imaging target [27–29]. A target consisting of only a black and white edge was imaged. The slope of the edge was set at 5° to give an optimal MTF, as demonstrated by Dumas et al [27]. A 128 by 64 pixel rectangle, consisting of equal parts black and white pixels, was selected from the edge line image for MTF calculations. The FOV of the system was determined by imaging a precision graded ruler, oriented horizontally and then vertically in relation to the imaging frame, from working distances of 20, 30 and 40 cm from lens to target. The FOV was found to increase in a linear fashion in both the horizontal and vertical directions (R^2^ > 0.999). Fluorescence imaging studies were conducted using various concentrations of the Indocyanine Green (ICG) (Sigma Aldrich, MO), dissolved in a Dimethyl Sulfoxide (DMSO) solvent. ICG has a peak excitation wavelength at 780 nm in DMSO and peak fluorescence emission at 830 nm. Serial dilutions of the ICG were made for goggle characterization studies and testing. The detection limits for fluorescence were determined by imaging various concentrations of ICG in DMSO. For this study, three different tubes filled with the fluorescent solution were imaged for each dye concentration. Empty tubes of the same variety were also imaged to ensure they contributed no autofluorescence, and tubes containing only DMSO were imaged to set the background level. The intensity of each fluorescent tube was determined to be the measured in 8-bits gray level intensity (measured from 0–255) of each tube minus the background intensity found from the DMSO tubes. The minimum detectable fluorescent intensity was defined as having a signal to background ratio (SBR) of at least 2.

Two non-fluorescing plastic tubes (5 mm Ø, 50 mm length) simulating blood vessels were filled with various concentrations of the ICG/DMSO solution. The tubes placed side by side were then imaged in order to determine the minimum resolvable distance in between the tubes that the system could detect for various dye concentrations. This was implemented at working distances of 20, 30 and 40 cm and using different ICG concentrations, based on the previously determined detection limits of the system. The distances used in between the tubes, as determined by precision calipers, were 0.25 mm, 0.64 mm and 1.27 mm. To further characterize goggle performance, chicken breasts were injected with a serial dilution of ICG in DMSO, in an array. Each injection contained 0.02 mL of solution and was administered into a small hole (0.5 mm Ø) made into the surface of the chicken, with the top portion exposed to the air. Syringes, each containing a different concentration of fluorescent solution, were prepared in advance, and imaging was conducted immediately following the final injection to avoid significant change in fluorescent concentration due to diffusion through the tissue.

The minimum resolvable depth perception provided by the stereoscopic imaging capability of goggles was estimated through experimental observations conducted by multiple users (n = 5). Stacks of varying quantities of 1 mm thick opaque glass slides were placed adjacent to each other, each stack containing between 1 and 4 slides. Each stack was assigned a number, and then two stacks were randomly selected and placed adjacent to each other. Two stacks containing the same number of slides could also be selected; this would function as a control for the experiment. Next, a randomly selected user would observe the adjacent stacks through the goggle while imaging the stacks from 20, 30, and 40 cm working distances. Finally, the user would indicate which stack was higher and by approximately what amount. The process was repeated until each stack combination was observed by each user.

### Image-Guided Surgery in Chicken Ex Vivo

Whole chickens were utilized to conduct surgeries guided by the Integrated Imaging Goggle system ex vivo. Three parallel experiments each for three different surgical studies were conducted, and signal to background statistics were derived. The first method involved implanting a 0.6 mL microcentrifuge tube containing 300 picomoles ICG and a 0.2 mL circular capsule containing 100 picomoles ICG under the skin of the chicken breast. The chicken was illuminated using our light source with and without the addition of unfiltered diffuse light, respectively. Image-guided surgeries were conducted using the 2-sensor setup integrated with the hand-held microscope. The goggle frames were displayed with the hand-held microscope image in picture-in-picture mode on the wearable display stereoscopically. Similar simulated surgeries were guided by the 4-sensor setup. The second set of surgeries involved implanting the fluorescent tube and capsule into the chicken breast at depths of about 3 mm under the top surface, with the skin in that region removed. Under illumination both with and without the addition of the unfiltered light components, the chicken was imaged using the 2-sensor setup and the ultrasound. The image from the ultrasound displayed with the goggle frames in picture-in-picture mode. The third surgery, under the same lighting conditions as the fluorescence-ultrasound study, again utilized the 2-sensor setup with the handheld microscope to guide a simulated tumor resection. In this case, 0.02 mL of 500 nM (11 picomoles ICG) of ICG/DMSO solution was injected directly into the skinless chicken breast at depths of 2–3 mm under the surface. The stereoscopic large FOV fluorescence imaging capability was utilized to assist in the removal of the fluorescent tissue, while the hand-held in vivo microscope was used in assessing the surgical margins for additional fluorescence. The individual in this manuscript has given written informed consent (as outlined in PLOS consent form) to publish these case details.

### Telemedicine

We demonstrated the feasibility of transmitting the images recorded from the goggle devices and streaming them in near real time to a remote viewer. To demonstrate the concept, we transmitted the real time video feed wirelessly to a remote viewer in a separate location through commercially available video streaming software. The remote viewer was then able to monitor the surgery in progress on a PC, laptop, tablet, smartphone, another set of Integrated Imaging Goggle or other internet ready mobile device. Our method was tested on WiFi as well as 4G LTE network. If the receiving end is another Integrated Imaging Goggle, the video stream can be viewed stereoscopically in 3D with depth perception.

## Results

### System Characterization

**Fig 2:**
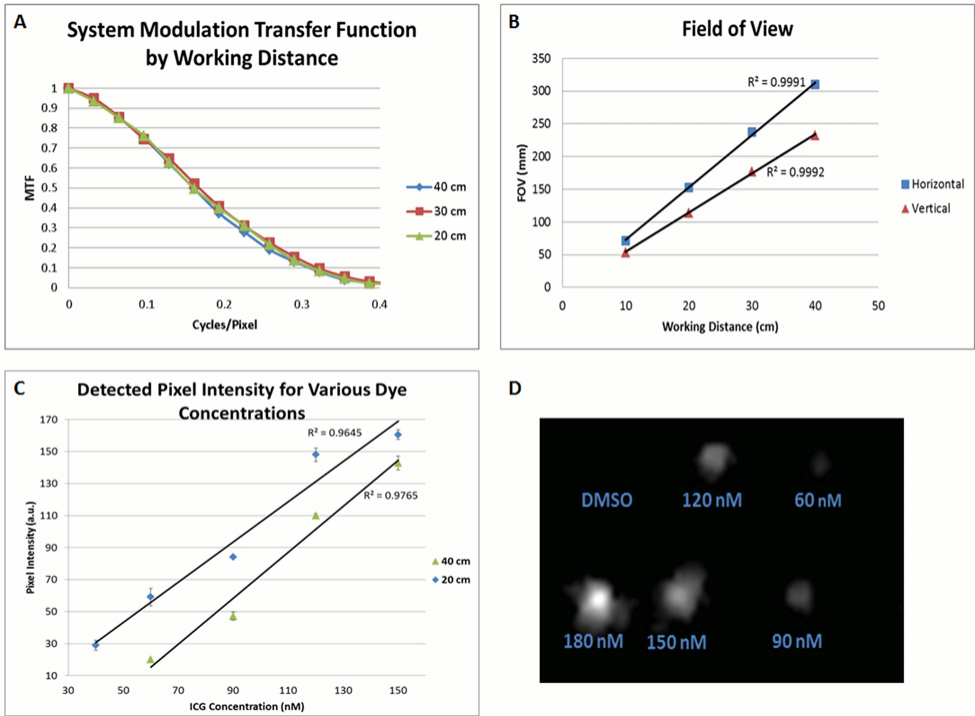
System Characterization. (A) Modulation transfer functions (MTF) for the NIR imaging detector as a function of cycles per pixel. (B) FOV measurements for the NIR imaging detector taken versus working distances of 20, 30, and 40 cm. Vertical and Horizontal FOV measurements were recorded. (C) Pixel intensity for the detected fluorescent emissions from excited solutions of ICG/DMSO of varying ICG concentration, as detected by the NIR imaging detector. The detected fluorescent intensity decreases with increasing working distance. In addition, the intensity of the fluorescence at any working distance increases linearly with dye concentration (R^2^ = 0.96). (D) The detected fluorescence from an injection of a serial dilution of ICG/DMSO into 0.5 mm holes cut into the surface of a chicken breast. Imaging was conducted at 40 cm working distance under 785 low pass filtered illumination.

To better understand the performance of wide FOV imaging capability offered by our system, we characterized the MTF of the Integrated Imaging Goggle using the slanted edge technique (**Fig 2A**). The results indicate the preferred transfer function, which is directly correlated to optimal resolution, at a working distance of 30 cm. The transfer function at working distances of 20, 30, 40 cm are very similar, indicating that the optimal working distance for the system lies within this range. The approximate working distance for a surgeon of typical height in operations is 30 cm ±10 cm. The field-of-view measurements taken from the wide-field fluorescence images of the goggle at 20, 30 and 40 cm working distances are shown in **Fig 2B**. The FOV increases linearly in both the vertical and horizontal directions with increasing working distance (R^2^ = 0.999).

Furthermore, the fluorescence detection limit of wide FOV imaging was also assessed (**Fig 2C**). As expected, the average detected fluorescent intensity increased with dye concentration for each working distance. The minimum detectable concentration over the background (SBR ≥ 2) was found to be 40 nM at the 20 cm working distance and 60 nM at 40 cm distance. The detected pixel intensity was found to increase with dye concentration in a linear fashion (R^2^ = 0.96). To test lateral resolution of the wide FOV fluorescence imaging, two fluorescent dye filled plastic tubes of various concentrations were utilized. After imaging the tubes side-by-side at various distances, it was determined that the wide FOV imaging can detect down to a 0.25 mm resolution at all working distances.

Chicken breast tissues were used to assess fluorescence detection in biological tissues for small volumes of ICG. The minimum concentrations detectable corresponded to the results from our three tube study: 40 nM (0.8 picomoles of ICG) at a 20 cm working distance, and 60 nM (1.2 picomoles ICG) at 30 and 40 cm working distances (**Fig 2D**). The fluorescence detection data obtained with chicken tissues are consistent with our data obtained with ICG-filled tubes.

The degree of stereoscopic vision provided by the Integrated Imaging Goggles was estimated by experimental observations. Users unanimously agreed that a 2 mm depth between adjacent slides was observable at any of our working distances through the goggles. Additionally, users reported an observable difference between adjacent slides for a 1 mm height difference at working distances less than or equal to 20 cm.

### Image-Guided Surgeries in Chicken

**Fig 3:**
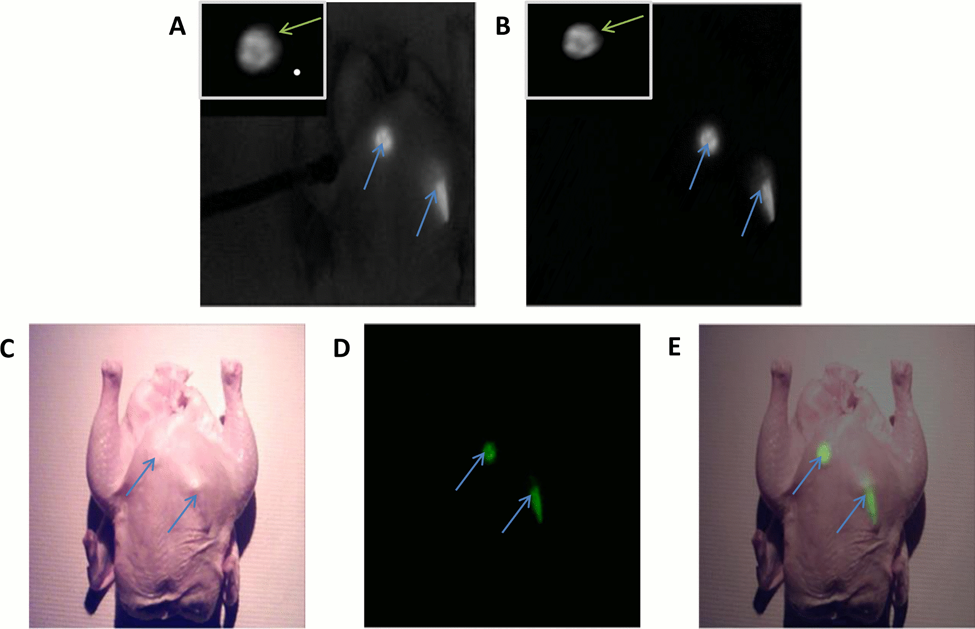
Image-guided surgeries aided by the Integrated Imaging Goggles. Intraoperative imaging using the 2-sensor setup (A) with the addition of unfiltered NIR light for the imaging of anatomical data, and (B) without unfiltered NIR light. Goggle aided stereoscopic imaging of 2 fluorescent targets (blue arrows) under the skin of a chicken. The image from the hand-held microscope is displayed in the top left corner of each frame from the large FOV imaging, displaying a view of the fluorescent targets with higher resolution (green arrows). (C) Intraoperative imaging using the 4-sensor setup: The anatomical information from the color reflectance imaging; (D) the functional information from fluorescence imaging. The detected fluorescence was pseudocolored in green to facilitate visualization; (E) the composite fluorescence and color reflectance images displayed to the user.

To better assess the performance of the Integrated Imaging Goggles, we performed image-guided surgeries in chicken, post mortem. Intraoperative imaging of the whole chicken with 2 fluorescent targets implanted beneath the skin was conducted using the Integrated Imaging Goggle (**Fig 3A and 3B**). This test was conducted with and without the addition of unfiltered NIR illumination from our light source and, under either lighting condition, the fluorescence was clearly visible above the background. The average SBRs over three trials were 5.8 ±0.18 and 134.8 ±7.1 for the surgeries with and without the addition of unfiltered NIR illumination, respectively. The wide FOV imaging guided the assessment of the larger area, while the hand-held microscope offered further investigation of lesions at higher resolution. The simulated lesions were clearly imaged and displayed stereoscopically by our system, in real time. It was found by the surgeon that the depth perception and stereoscopic imaging capabilities are crucial for guiding surgeries and help to improve hand-eye coordination. The hand-held microscope also augmented the assessment of simulated lesions.

Imaging of the chicken with the same 2 fluorescent targets implanted beneath the skin was conducted using the 4-sensor setup (**Fig 3C–3E**). The 4-sensor setup allowed us to attain both anatomical information from color reflectance imaging (**Fig 3C**), as well as functional information from fluorescence imaging (**Fig 3D**), in real time. The fluorescence data was presented to user in a pseudocolor (green) and then merged with the anatomical data into a combined imaging frame (**Fig 3E**). Combining the functional and anatomical data allowed the surgeon to better localize the fluorescence information with respect to the background, potentially improving surgical outcomes. The margin of error for registering the fluorescent data to the anatomical data was determined to be less than 1 mm in any direction when the goggle was used in the typical working distances (20–40 cm), following the calibration procedures. A calibration correction metric was used every 4 cm within the calibrated working distance (i.e. one correction metric at 20 cm, another at 24 cm, etc.), providing a continuous registration with error less than 1 mm. Error was determined by comparing the locations of the calibration targets in the overlaid image.

**Fig 4:**
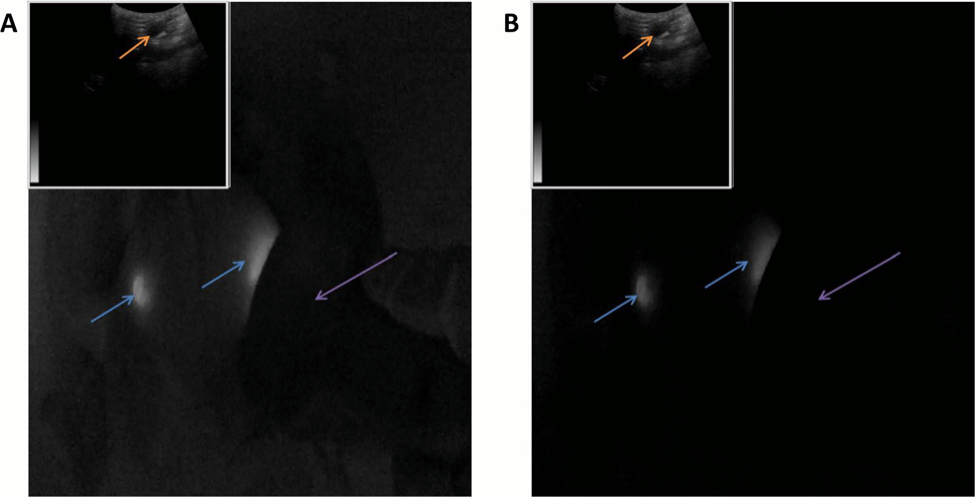
Goggle aided stereoscopic imaging with ultrasound. 2 fluorescent targets (blue arrows) implanted into the chicken breast at depths of approximately 3 mm. Ultrasound imagery is displayed in picture-in-picture mode at the upper left of the goggle imaging frame. The ultrasound transducer (purple arrow) was capable of detecting the implanted fluorescent tube as a dark region (orange arrow), similar to a large vessel or fluid filled sac. Imaging was conducted with (A) and without (B) unfiltered NIR illumination.

The second surgical study was conducted using the goggle system integrated with ultrasound, displayed onto the stereoscopic goggle frames in picture-in-picture mode (**Fig 4**). The chicken was illuminated with and without unfiltered NIR light. Fluorescent targets were clearly visible above the background in the large FOV images under both lighting conditions; averaged (n = 6) SBR was 442 ±21 without unfiltered NIR light and 5.4 ±0.12 with the unfiltered NIR light, respectively. Ultrasound was able to detect the implanted fluorescent tube which appeared as a hypoechoic dark, elongated object simulating a large blood vessel or fluid filled sac. The ultrasound images provide information complementary to fluorescence images. The depth penetration of ultrasound imaging is also desirable for deeper tissue assessment, reported by the surgeon.

**Fig 5:**
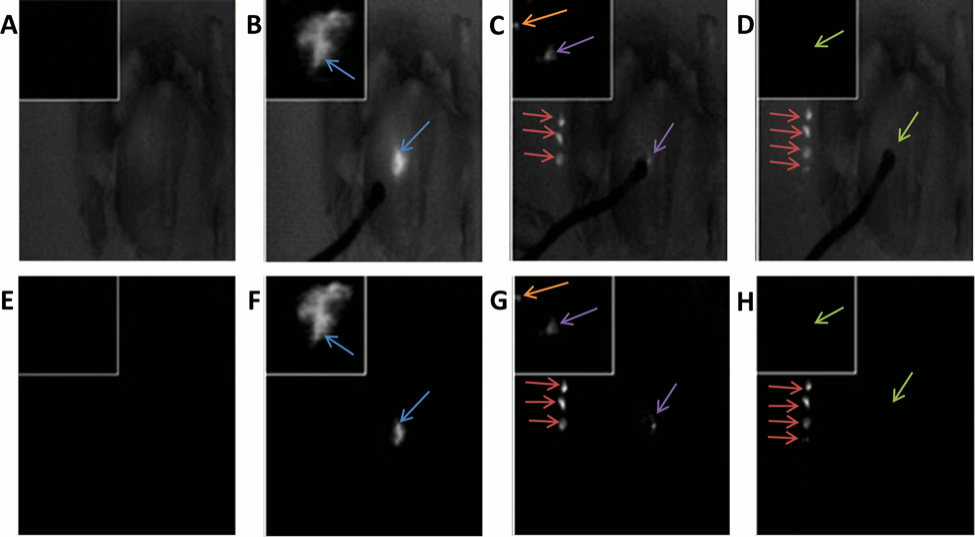
The surgical resection of fluorescent tissues in chicken guided by the Integrated Imaging Goggle with its hand-held microscope. High-resolution microscopic images were incorporated in picture-in-picture mode into the wide FOV stereoscopic goggle frames. Images displayed were illuminated using our light source with unfiltered NIR components for anatomical data (A-D) and using only the low pass filtered light (E-H). The images are from four distinct time points during the resection of the fluorescent tissue: (A & E) Chicken pre-injection; (B & F) post-injection and prior to any resection; (C & G) after a partial resection (excised tissue indicated by red arrows), note the residual lesions in both the goggle and microscope images (purple arrows); orange arrows indicate small residual lesions that are only revealed by the microscopic imaging; and (D & H) after complete resection and removal of residual fluorescent tissues (green arrows).

The third surgical study performed involved injecting the chicken breast with fluorescent dye, and conducting a resection of the fluorescent tissue guided by the Integrated Imaging Goggles with its hand-held microscope. Under illumination with and without the unfiltered NIR light, the chicken was imaged throughout the surgical procedure using the goggle (**Fig 5**). The simulated lesion exhibited higher florescence intensity over the background under both illumination regimes (**Fig 5B and 5F**). After the partial resection of the simulated lesion, residual fluorescent tissue was still visible around the incision site (**Fig 5C and 5G**). The average (n = 3) SBR between the simulated cancer lesions and the control breast tissues illuminated by our light source with unfiltered NIR lighting included was 3.94 ±0.04. The SBR using only the 785 nm low pass filtered illumination was found to be 220 ±14. All three tests were conducted using the same illumination intensity as well as the same distance from the light source to the target. The hand-held microscope provided an enhanced view of the surgical margin status with high resolution. The high resolution of the hand-held microscope complemented the large field-of-view of the stereoscopic fluorescence imaging, facilitating surgical decision making. These studies have demonstrated the potential value of the Integrated Imaging Goggle system in assisting with surgical resections.

We have included a supplementary video in which the Integrated Imaging Goggles provide intraoperative imaging in a simulated surgery in chicken (S1 Video). Also demonstrated in the video are 6 different viewing modes, including various picture-in-picture and single-frame displays.

### Telemedicine

**Fig 6:**
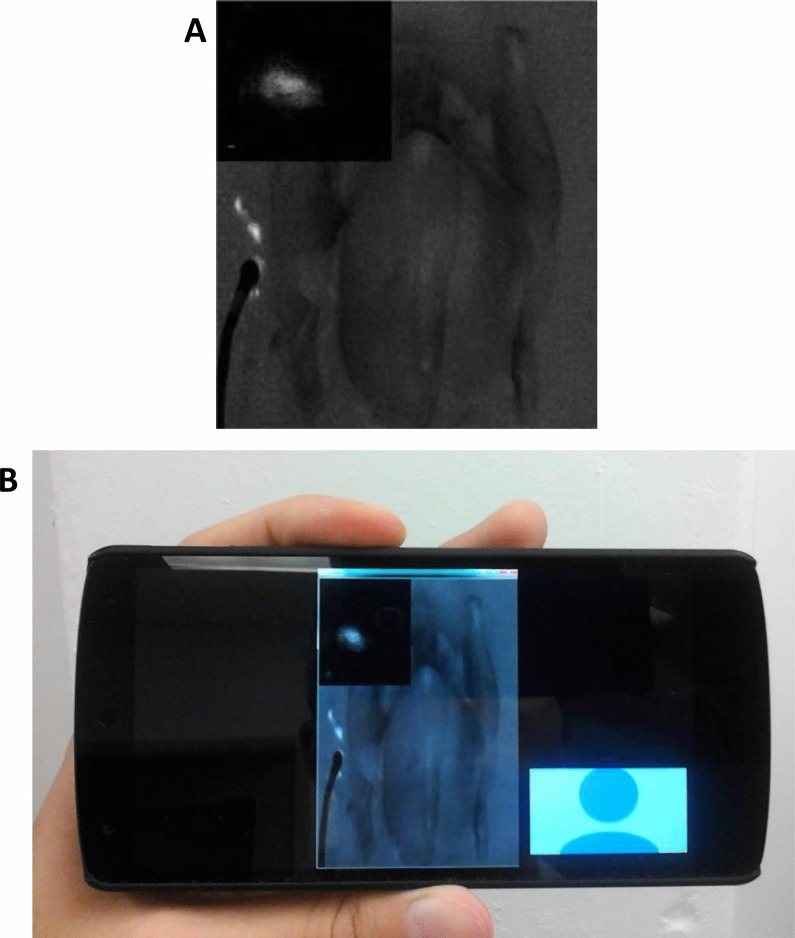
Telemedicine enabled by the Integrated Imaging Goggle. (A) The fluorescence video stream as seen through the remote goggle, transmitted via 4G LTE network. Orange arrows indicate simulated cancerous lesions. (B) The received fluorescence video frame displayed on the remote viewer’s smartphone on the go. Red arrows indicate simulated cancerous lesions.

One important feature of the Integrated Imaging Goggles is the wireless connectivity that facilitates telemedicine and remote collaboration. The real time video from the goggle was shared over the internet on WiFi as well as 4G LTE network to a remote location (delay < 0.1 second). For this experiment, we demonstrated the feasibility of wirelessly transmitting video from the goggle to another goggle, a smartphone or a computer. The image as seen through the remote goggles is shown in (**Fig 6A**). When another set of Integrated Imaging Goggle is used, we were able to view the image data stereoscopically in 3D at the remote site. Therefore, we have demonstrated goggle-to-goggle stereoscopic video capture, transmission and display, which facilitate the assessment of surgical scene from remote site with the depth perception. A video captured by the goggle detectors could also be transmitted to the remote viewer to be viewed on a mobile device such as a smartphone or a tablet computer (**Fig 6B**).

## Discussion

In this paper, we reported the development of Integrated Imaging Goggle systems that offer both wide-field fluorescence imaging and hand held microscopic imaging to guide surgery. Ultrasound imaging is also integrated into the goggle system. It is the first wearable real time stereoscopic fluorescence imaging system with large FOV and depth perception. The real time registration of color reflectance images and fluorescence images are also desirable, to provide both functional information and structural information.

Implementing a stereoscopic 3D imaging system can significantly benefit tumor resections and SLN mapping in real time. Stereoscopic or binocular vision allows for significantly improved depth perception. This occurs via the disparity between objects located in images as seen by our right versus left eyes. The brain processes this disparity, or parallax, into depth information [6]; for example, this is how a surgeon knows when to initiate or stop a surgical task such as an incision. This information cannot be accurately attained from a 2D image alone [6]. In medical applications, stereoscopic information can help a surgeon distinguish the 3 dimensional shape of a lymph node or the depth of a small tumor on the surface of an organ. In addition, faster surgical completion times have been reported when using stereoscopic display systems [30]. Previous research into 3D medical imaging has found applications in ophthalmology, mammography, oncology, orthopedic studies, and vascular mapping among others [6, 30–31]. Various modalities have been implemented for 3D stereoscopic imaging including X-ray, CT and visual imaging [32–36]. In detecting breast cancer lesions, Getty et al found a 44% decrease in false positives when utilizing stereoscopic mammography [37]. Results such as these demonstrate a potential advantage in having depth perception for medical imaging.

In characterizing our system, we have determined sub-millimetric optical resolution with a large FOV when utilizing either reflectance mode or fluorescent imaging. In addition the fluorescent detection limit for our system was found to be 40–60 nM range. These factors are important when detecting small residual tumors off of the main lesion site as well as when accurately mapping the tumor margins or SLN locations. Our system is very sensitive and can detect 90 picomoles of ICG. While other imaging systems with a larger size may offer a higher level of fluorescent detection sensitivity, our device trades off a small degree of sensitivity to achieve a light weight and compact design, given ergonomics is very important for a wearable system. Additionally, it should be noted that the fluorescent detection limits are not fixed, and can be varied by changing the working distance of the cameras to the fluorescent target as well as by altering the intensity of the excitation light. Previous studies on liver cancer imaging in humans used ICG (0.5 mg/kg body weight) which is equivalent to 4.5 micromoles of ICG for a patient weighing 75kg [24, 38]. In another clinical study 0.5 mM of ICG was used for sentinel lymph node mapping [39]. Given the parameters reported in these published studies [24, 38, 39], we think the detection sensitivity of our system, 60nM/90 picomoles, can potentially serve these surgical oncologic applications well. In the future we will verify the detection sensitivity in clinical studies.

To further improve the system’s ability to detect small residual tumors, we have incorporated a hand-held fluorescence microscope which is displayed using picture-in-picture mode. Picture-in-picture format provides the user with a convenient means of visualizing the microscopic (and also ultrasound) data without losing sight of the wide FOV fluorescent imaging data, and without compromising the stereoscopic effect of the 3D display. Surgeons appreciate this feature as it preserves the line-of-sight fluorescence imaging while integrating additional imaging functionality. The intuitive nature and conveniences offered by the Integrated Imaging Goggle has potential to reduce the training curve and expedite surgical procedures.

In addition to a small handheld microscope, we have integrated a portable ultrasound into the goggle system, making it a platform technology for multimodal imaging. The benefit of ultrasound incorporation is to provide complementary information and further improve visualization of depth information, beyond the depth penetration of optical imaging. Depending on the frequency of the ultrasound transducer, the typical maximum depth penetration through soft tissues is approximately 20 cm for deep region scanning (3–5 MHz) between 5 cm (1 MHz) and 2.5 cm (3 MHz) [40]. Depth penetration of light from infrared fluorophores can vary depending on the dye concentration, detector sensitivity, process of detection, working distance and illumination intensity. Typical reports on maximum penetration depth range is approximately 2 cm, depending on the tissue types [41–42]. Ultrasound complements the surface-weighted functional information provided by fluorescence with additional structural information at various depths through the tissue. Visualization of ultrasound data in picture-in-picture format provides the surgeon with simultaneous line-of-sight, stereoscopic fluorescence imaging as well as structural ultrasound data in real time.

We have conducted real-time wireless communications with our system, transmitting from the surgical side goggle system to a remote site PC, goggle or mobile device. The communication protocols effectively enabled goggle-to-goggle video transmission for the first time, allowing the remote viewer to visualize the surgical landscape stereoscopically in 3D which was previously unavailable. This method of sharing real time medical data can allow for a surgeon to collaborate with other clinicians when making time sensitive decisions during a surgical intervention. In addition, herein we also reported the first demonstration of goggle-to-smartphone telemedical collaboration. The possibility to enable collaboration with various mobile devices opens the door to new solutions of medical logistics management. Consequently, the workflow can be improved and team collaboration can be facilitated. The compact size, light weight and relative lower cost of the Integrated Imaging System make it an appealing option to clinicians in a variety of clinical settings.

### Future Work

Our current prototype incorporates a hand-held fluorescence microscope with VGA resolution. While this is good for demonstrating the concept, another hand-held microscope with a higher resolution sensor and a higher power lens may be developed in the future, providing us with a higher resolution and improved image quality for better tumor delineation and localization. Also, the registration accuracy of the 4-sensor setup may be further improved in future iterations by further development of registration algorithm. To date, we have characterized the system and conducted surgical studies on biological tissues post mortem or ex vivo. In the future we will conduct goggle-aided image guided surgeries in rodents and large animals, with tumor models simulating various human cancers. While herein FDA-approved tracer ICG is used as the tracer to demonstrate the translational potential, in the future we will explore the preclinical molecular-targeting tracers for ideal pharmacokinetics and biodistribution.

Previously we have contributed to surgical innovations including (a) America's first near total face transplant [43], (b) the maxillary approach for neuromodulation of sphenopalatine ganglion and cluster/migraine headache, (c) fulcrum spreader graft for nasal valve repair, and (d) the bone anchored facial suspension for facial nerve paralysis. In the future we also plan to explore the potential of the Integrated Imaging Goggle in facilitating these surgical procedures.

## Conclusions

In summary, we have developed novel Integrated Imaging Goggles for guiding surgeries. The prototype systems offer real time stereoscopic wide-field fluorescence imaging and color reflectance imaging capacity, along with in vivo handheld microscopy and ultrasound imaging. The goggle-to-goggle and goggle-to-smartphone telemedicine are also enabled to facilitate collaboration. The Integrated Imaging Goggles have shown great potential for facilitating image-guided surgeries.

## Supporting Information

S1 Video Supporting video of using Integrated Imaging Goggles. The Integrated Imaging Goggles provide intraoperative imaging in a simulated surgery in chicken. Also demonstrated in the video are 6 different viewing modes, including various picture-in-picture and single-frame displays.
(MP4)
